# Discrimination and Characterization of the Aroma Profile in Four Strawberry Varieties Cultivated Under Substrates

**DOI:** 10.3390/foods14091464

**Published:** 2025-04-23

**Authors:** Su Xu, Dajuan Shi, Fengwei Ma, Guangcan Tao, Jieling Xu, Lingshuai Meng, Haijiang Chen, Sen Cao, Dong Lin, Qiang Fei, Yi Liu, Siyao Wu

**Affiliations:** 1 Guizhou Engineering Research Center for Characteristic Flavor Perception and Quality Control of Dual-Food Homologous Resources, College of Food Science and Engineering, Guiyang University, Guiyang 550005, China; dj99102023@126.com (D.S.); mfw200422501212@163.com (F.M.); tgcan@126.com (G.T.); 15040260380@163.com (L.M.); b05chenhj@126.com (H.C.); cs5638myself@126.com (S.C.); gyulindong@163.com (D.L.); fqorganic@163.com (Q.F.); ceci41@126.com (S.W.); 2 College of Biology and Environmental Engineering, Guiyang University, Guiyang 550005, China; catcattaotao@163.com; 3 Guizhou Vocational College of Foodstuff Engineering, Guiyang 551400, China; 18010148728@163.com

**Keywords:** strawberry fruit, volatile organic compounds, biomarkers, HS-GC-IMS, E-nose

## Abstract

The strawberry is renowned for its distinctive fragrance and is regarded as one of the most popular fruits globally. This research analyzed the volatile compounds in four strawberry types grown in substrate systems, utilizing HS-GC-IMS, HS-SPME-GC-MS, and E-nose techniques. The results revealed a notable increase in the relative concentrations of alcohols, esters, and aldehydes in the strawberries. The E-nose was able to differentiate between the various strawberry varieties, but it was unable to fully identify specific aroma compounds. In contrast, the HS-GC-IMS and HS-SPME-GC-MS techniques demonstrated effectiveness in distinguishing and characterizing the different strawberry types, with OPLS-DA employed for further evaluation. By applying the variable importance in projection (VIP) method, six and seven aroma components were identified as potential biomarkers by GC-MS and GC-IMS, respectively. This study lays a scientific foundation for identifying key aromatic components in strawberries grown via substrate cultivation and offers comprehensive insights into their aromatic properties.

## 1. Introduction

Strawberry (*Fragaria ananassa* Duch.) is renowned for its distinctive aroma and ranks among the most popular fruits globally [1]. While strawberries are predominantly consumed fresh, they are also frequently utilized in the production of juices, jams, and ice cream [2,3]. As a non-climacteric fruit, the aromatic qualities of strawberries are permanently established once they are harvested [4]. With the expansion of the strawberry economy and evolving consumer expectations regarding fruit quality, researchers have shifted their focus beyond basic quality metrics, such as yield and general fruit quality, to include the aroma of strawberries [5]. Strawberries exhibit one of the richest diversities of aromatic substances among fruits, making aroma a critical component of strawberry fruit [1]. Although the volatile organic compounds (VOCs) in fresh strawberries comprise only 0.001% to 0.01% of their total composition, they are essential in the development of strawberry aroma profiles [6]. A total of 360 VOCs has been observed in connection with the aroma of strawberries, primarily comprising furans, esters, terpenes, alcohols, aldehydes, ketones, and sulfides [6,7,8]. Among these, 2,5-dimethyl-4-hydroxy-3(2H)-furanone (DMHF) and 2,5-dimethyl-4-methoxy-3(2H)-furanone (DMMF) are regarded as the primary VOCs contributing to strawberry aroma [9,10]. The distinct aroma of strawberry fruit serves as a clear example of the importance of aroma, as different strawberry varieties possess unique VOCs that result in specific aromas for each variety.

In recent years, the aging demographic of strawberry growers and rising labor costs have led to the emergence of soilless cultivation, both domestically and internationally. Innovative methods, such as substrate cultivation, have gradually replaced traditional soil cultivation techniques and are emerging as a new trend in strawberry farming [11]. This shift is attracting increasing attention from both growers and consumers. González-Domínguez et al. [12] employed headspace solid-phase microextraction gas chromatography–mass spectrometry (HS-SPME-GC-MS) to assess the distinctive VOCs found in three types of strawberry fruits, Festival, Candonga, and Camarosa, grown in a soilless cultivation environment. The findings revealed that the Festival and Candonga varieties exhibited a rich diversity and high content of aromatic substances, in particular, elevated levels of furanones, esters, and terpenes. Liu et al. [13] utilized HS-SPME-GC-MS technique to examine the VOCs present in four strawberry varieties (Benihoppe, Tochiotome, Sachinoka, and Guimeiren) under soilless cultivation conditions. Among these varieties, 37, 48, 65, and 74 VOCs were determined, predominantly comprising esters, aldehydes, and terpenes.

The scent of strawberries grown in soilless systems utilizing substrates is greatly shaped by the different varieties of strawberries and subsequently impacts consumer choices [11]. Therefore, it is essential to investigate the volatile aromas of various strawberry varieties grown in such systems to specify the typical VOCs included in strawberries. In typical circumstances, earlier investigations mainly employed HS-SPME-GC-MS, gas chromatography–olfactometry–mass spectrometry (GC-O-MS), and headspace gas chromatography–ion mobility spectrometry (HS-GC-IMS) for the detection and measurement of VOCs, in addition to auxiliary electronic nose (E-nose) techniques [14]. The electronic nose is a powerful electromechanical sensory device that enables the discrimination of aroma profiles among various strawberry variety matrices; however, it does not provide the specific compounds that determine organoleptic quality [15]. In contrast, HS-SPME-GC-MS can address this limitation due to its ability to identify key aroma-active compounds [16,17]. Furthermore, HS-GC-IMS has become increasingly favored for identifying isomeric VOCs, such as those that exist as ring isomers [18,19]. This technology does not require SPME to extract VOC concentrations from the sample; instead, it can directly extract and analyze specific amounts of gases from the sample [20]. This method not only promises a more precise representation of a sample’s actual aroma profile but is also quicker, more sensitive, and more cost-effective compared to GC-MS [21].

Therefore, this study aimed to assess the VOCs in four types of strawberries, ‘Hongyan, HY’, ‘Fenyu, FY’, ‘Xiangye, XY’, and ‘Jiandehong, JDH’, cultivated under uniform substrate conditions, utilizing HS-GC-IMS, HS-SPME-GC-MS, and E-nose techniques. To evaluate the efficacy of these three techniques for sample detection and identification, statistical approaches such as orthogonal partial least squares discriminant analysis (OPLS-DA) and principal component analysis (PCA) were utilized. Additionally, the variable importance in projection (VIP) method was utilized to identify key aroma biomarkers across four fresh strawberry varieties. Analyzing VOCs under substrate-based soilless cultivation conditions for these four different strawberry varieties will aid in evaluating strawberry quality and guiding consumer purchases.

## 2. Materials and Methods

### 2.1. Sampling

Four varieties of strawberries—specifically, ‘Hongyan HY’, ‘Fenyu FY’, ‘Xiangye XY’, and ‘Jiandehong JDH’—were cultivated in a greenhouse operated by Guizhou TSQF Ecological Agriculture Development Co., Ltd. (Guiyang, China), located in the Huaxi district of southwestern China (latitude 26°45′ N, longitude 106°62′ E, elevation 1090.8 m, average annual temperature of 15.7 °C, average yearly rainfall of 1469.6 mm, and average relative humidity of 77%) (Figure S1). The fruits from these strawberry cultivars were harvested from 20 to 25 December 2023. All selected varieties were grown in the same soilless substrate under controlled greenhouse conditions, receiving uniform drip fertilization through a shared pipe to ensure consistent planting conditions. The substrate comprised a mixture of coir, peat, and perlite in the proportions of 5:3:2 [16]. A vertical iron frame, positioned 1.1 m above the ground, supported a strawberry cultivation trough. The distance between each strawberry plant was maintained at 30 cm, while the spacing between rows was 35 cm, allowing for the cultivation of approximately 6800 plants within an area of 667 m^2^.

The methods for preparing fertilizer were adapted from the approaches outlined in [22,23], with necessary adjustments made to accommodate the unique conditions of the base. During the vegetative phase, the levels of essential nutrients were recorded as follows: nitrogen (N) at 193.32 mg/L, phosphorus (P) at 72.94 mg/L, potassium (K) at 215.32 mg/L, calcium (Ca) at 78.05 mg/L, magnesium (Mg) at 40.00 mg/L, iron (Fe) at 6.16 mg/L, boron (B) at 0.53 mg/L, zinc (Zn) at 0.04 mg/L, manganese (Mn) at 0.04 mg/L, copper (Cu) at 0.02 mg/L, and molybdenum (Mo) at 0.01 mg/L. In the fruiting phase, the concentrations of nutrients were recorded as follows: nitrogen (N) measured 144.45 mg/L, phosphorus (P) at 39.09 mg/L, potassium (K) at 311.29 mg/L, calcium (Ca) at 41.70 mg/L, magnesium (Mg) at 34.47 mg/L, iron (Fe) at 6.13 mg/L, boron (B) at 0.40 mg/L, zinc (Zn) at 0.04 mg/L, manganese (Mn) at 0.03 mg/L, copper (Cu) at 0.01 mg/L, and molybdenum (Mo) also at 0.01 mg/L. Furthermore, the pH levels were meticulously regulated to remain within the optimal range of 5.8, while the electrical conductivity (EC) was maintained between 1.2 and 1.7 mS/cm.

### 2.2. Reagents and Instruments

A standard comprising n-alkanes ranging from C_7_ to C_40_, which was employed in gas chromatography–mass spectrometry analysis, was obtained from Amprite Standard Technical Service Co., Ltd., located in Shanghai, China. The analysis of volatile substances covered a broad range, from C_7_ to C_40_, encompassing compounds with low boiling points (e.g., C_7_, boiling point ~98 °C) and those with high boiling points (e.g., C_40_, boiling point ~450 °C), thereby facilitating retention index calibration for most volatile and semi-volatile organic compounds. Additionally, the retention indexes of numerous compounds in prominent mass spectrometry libraries, such as NIST, were based on n-alkanes; employing the same standards could enhance the identification accuracy. Furthermore, normal alkanes possessed stable chemical properties and exhibited minimal reactivity with the analyte, rendering them suitable as inert reference materials. The E-nose (PEN 3) was provided by Airsense Analytical GmbH in Germany. The 7890-5975C GC-MS was from Agilent Corporation in Santa Clara, CA, USA. Finally, G.A.S Inc. in Seevetal, Germany, supplied the FlavourSpec GC-IMS.

### 2.3. Volatile Aroma Profile Evaluation

#### 2.3.1. Electronic Nose Evaluation

The aroma profiles of four distinct strawberry varieties were systematically evaluated using PEN 3 electronic nose equipment, which was manufactured by Airsense Analytics Co., Ltd., based in Schwerin, Germany. This sophisticated system featured ten metal oxide sensors, each meticulously calibrated to detect specific volatile compounds such as aromatic benzene, nitrogen oxides, ammonia, hydrogen, alkane aromatic compounds, short-chain alkanes, sulfides, terpenes, alcohols, aldehydes, ketones, organic sulfides, and long-chain alkanes [14]. In the experimental procedure, 2 g of each strawberry sample was carefully transferred into a 20 mL GC vial and incubated at room temperature for a duration of five minutes prior to conducting the measurements. The parameters for detection were rigorously defined and included a sample measurement time of 120 s, a cleaning interval of 100 s, a pre-injection phase lasting 5 s, and a reset period of another 5 s. The injection flow rates and carrier gas were set at 200 mL/min to ensure effective analysis. The data obtained from these measurements were then subjected to principal component analysis (PCA), which was performed using the software (Winmuster) integrated into the electronic nose system, allowing for an insightful examination of the aroma profiles.

#### 2.3.2. HS-GC-IMS Evaluation

The GC-IMS instrument utilized for the study was the FlavourSpec^®^, developed by G.A.S. Gesellschaft für analytische Sensorsysteme mbH, located in Dortmund, Germany. This sophisticated apparatus featured a CTC-PAL 3 headspace automatic sampler from CTC Analytics AG Co., Ltd., based in Zwingen, Switzerland, which facilitated the efficient collection of VOCs from the samples being analyzed. In this specific experiment, a 5 g sample of strawberries was transferred into a 20 mL glass vial and subjected to an incubation process at a controlled temperature of 50 °C for a duration of 30 min. During the headspace extraction, a volume of 500 µL was injected at a speed of 500 revolutions per minute while the injection syringe was kept at a temperature of 85 °C to ensure the efficient volatilization of the compounds. In order to prepare the VOCs for analysis, they were pre-separated, employing an MXT-5 capillary column, which measured 15 m in length and had a diameter of 0.53 mm with a film thickness of 1.0 μm, supplied by Restek in the USA. The column temperature was held steady at 60 °C, and pure nitrogen, characterized by its high purity of 99.999%, was employed as the carrier gas. The flow rate of the carrier gas was initially set to 2 mL/min for the first 2 min and was then increased linearly to reach 10 mL/min over the course of the next 8 min. Following that period, the flow rate escalated further to 100 mL/min within 10 min and was sustained for an additional 20 min. The entire chromatographic run lasted for a total of 40 min, with the inlet temperature maintained at 80 °C to enhance the vaporization of the samples.

In the analysis conducted using IMS, a tritium ionization source, denoted as 3H, was utilized. The setup included a migration tube measuring 53 mm in length, through which ions were analyzed. An electric field intensity was established at a level of 500 V/cm to facilitate the movement of ions through the migration tube. Furthermore, the tube was maintained at a controlled temperature of 45 °C to optimize the ion mobility process. The drift gas employed in this analysis was high-purity nitrogen and possessed an exceptional purity level of 99.999%. This nitrogen gas was allowed to flow at a consistent rate of 75.0 mL/min while operating in positive-ion mode. To identify the VOCs present, the retention index for each target substance was calculated based on the specific retention time of that substance within the migration tube. This methodology enabled the accurate assessment and identification of various VOCs by leveraging the unique retention characteristics associated with each compound. These data were then compared against the retention indices of standard substances cataloged within the GC-IMS library provided by G.A.S.

For qualitative analysis, VOCal software was utilized, which incorporated built-in databases of GC retention indices (NIST 2020) and IMS migration times. Additional analytical comparisons among VOCs from different samples were performed using various plugins within the VOCal software, including Reporter, Gallery Plot, and Dynamic PCA. This enabled researchers to generate diverse analytical representations such as three-dimensional and two-dimensional spectra, distinctive fingerprints, and PCA plots for a comprehensive comparation of the VOCs present in the strawberries.

#### 2.3.3. HS-SPME-GC-MS Evaluation

The strawberry fruit was homogenized using a mortar to create a uniform mixture. Five grams of strawberry mixtures was carefully measured and placed into a 20 mL GC vial. To this vial, 10 μL of a standard solution of ethyl phenylacetate, which had a concentration of 8.21 mg/L, was added. The vial was then securely sealed using a screw cap that was equipped with a PTFE–silicon spacer, ensuring that the internal environment remained stable. This prepared vial was subsequently positioned on a thermostatic magnetic stirrer set to maintain a temperature of 50 °C for a duration of 15 min. Following this initial mixing period, a DVB/CAR/PDMS solid-phase microextraction needle, characterized by a film thickness of 50/30 µm and a length of 2 cm, was inserted into the vial. This needle was used to absorb VOCs from the sample for a period of 30 min, while maintaining the temperature at 50 °C. After the absorption process was complete, a desorption phase was initiated, involving a temperature setting of 250 °C for 3 min to retrieve the absorbed VOCs for subsequent analysis.

A GC-MS system, specifically the 7890-5975C model from Agilent Corporation in Santa Clara, CA, USA, was utilized for the separation and analysis of VOCs found in strawberry fruit samples. This advanced analytical technique was facilitated by an HP-5 MS column with dimensions of 60 m in length, 250 μm in internal diameter, and a film thickness of 0.25 μm, also provided by Agilent. The temperature programming of the GC oven was meticulously controlled to optimize the separation process. It commenced at an initial temperature of 40 °C, which was maintained for a duration of 3 min. Following this, the temperature was gradually increased to 80 °C at a rate of 5 degrees Celsius per minute. This was succeeded by a more rapid ramp up to 160 °C at a rate of 10 °C per minute, followed by a hold time of 0.5 min. The temperature was then further increased to 175 °C at a rate of 2 °C per minute and subsequently raised to 230 °C at a rate of 10 °C per minute, where it was held for a final period of 7 min. The analysis proceeded under splitless mode, with the carrier gas pressure precisely set at 103 kPa to ensure optimal flow conditions. The electron ionization (EI) mode was employed at an energy level of 70 eV to facilitate the ionization of the VOCs for mass spectrometric detection. To maintain the integrity of the analysis, specific operational parameters were configured; the temperature of the quadrupole was set to 150 °C, while the ion source temperature was maintained at 230 °C. Additionally, a solvent delay time of 7 min was implemented to effectively reduce the interference from the solvent in the initial phases of the analysis. Throughout the experiment, full scan mode was executed across a mass-to-charge ratio (*m*/*z*) range of 45 to 500, allowing for comprehensive detection and identification of the various compounds present in the strawberry fruit samples.

To identify VOCs in strawberry fruit samples, two distinct methods were implemented. The first approach involved the use of the MassHunter database integrated into the Agilent mass spectrometry workstation, which facilitated a thorough comparison of the detected compounds. Following this initial analysis, retention index (RI) values were calculated using a series of standards ranging from C_7_ to C_40_. These calculated RI values were then cross-referenced with data from the NIST Chemistry WebBook database, accessible at webbook.nist.gov, allowing for a robust verification of compound identities. To quantify the relative contents of the VOCs present in the samples, the peak areas of the identified VOCs were compared against those of internal standards (phenylethyl acetate), enabling a precise measurement of each compound’s concentration within the strawberry samples.

$$The\;relative\;content\;of\;VOCs\;\left(µg/g\right)=\frac{Peak\;area\;of\;VOCs\times Amount\;of\;internal\;standard\;(µg)}{Peak\;area\;of\;internal\;standard\times Dry\;weight\;of\;strawberry\;fruit\;(g)}$$

### 2.4. Relative Odor Activity Value (ROAV) Analysis

Relative odor activity value (ROAV) analysis is a method for assessing the impact of VOCs on the overall odor profile of a strawberry sample. This value is derived from the relationship between the relative concentration of VOCs in water and their respective odor thresholds (OTs) [24]. VOCs with an ROAV greater than 1 are classified as aroma components and have a significant influence on the aroma of strawberry fruits [25]. The formula used to calculate ROAV is as follows:

*ROAV_i_* = *C_i_*/*OT_i_*

where *C_i_* (mg/kg) refers to the relative levels of VOCs, and *OT_i_* (mg/kg) represents the odor thresholds of VOCs in water.

### 2.5. Data Analysis

The findings were presented as means ± standard deviation (SD). Significant differences were determined using IBM SPSS Statistics 25.0 (SPSS Inc., Chicago, IL, USA). Figure creation was accomplished with Origin 2021 (Origin Lab Corporation, Northampton, MA, USA). The VOC heatmap was generated using TBtools (version 0.655). Principal component analysis (PCA) and orthogonal partial least squares discriminant analysis (OPLS-DA) were performed with the Swedish software SIMCA (version 14.10, Umeå, Sweden).

## 3. Results and Discussion

### 3.1. E-Nose Analysis in Four Strawberry Fruit Varieties

**Figure 1:**
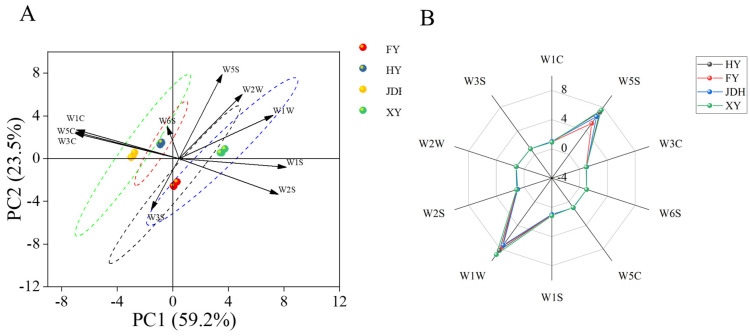
E-nose diagrams for the four types of strawberry fruits. (**A**) Biplot; (**B**) radar chart.

The E-nose has been extensively proposed for assessing food quality, utilizing devices that consist of arrays of gas sensors specifically designed for the selective measurement of components present in samples [26]. Principal component analysis (PCA) is a widely recognized unsupervised technology frequently employed to uncover hidden information within datasets through a reduced number of variables known as principal components [27]. For this research, Figure 1A illustrates the clustering results of various sample groups based on PCA. The contribution rates of PC1 and PC2 were 59.2% and 23.5%, respectively, yielding a cumulative explained variance of 82.7%. This indicated that these two components effectively captured the overall variation trend in the data. The spatial distribution of samples in the PCA space correlated with their similarity in principal component features; samples located closer together exhibited greater similarity in the characteristics represented by PC1 and PC2. The analysis revealed significant differences in the composition of VOCs among the four types of strawberries, allowing for clear differentiation. The arrows in Figure 1A represent the eigenvectors of each variable, with their lengths reflecting the contribution of each variable to the principal components. A smaller angle between the arrow and the coordinate axis indicates a stronger correlation between the variable and the corresponding principal component. Specifically, XY is situated in the first quadrant and exhibits a strong correlation with W1W and W1S, suggesting a significant response to sulfides and methane compounds. In contrast, HY and JDH are positioned in the second quadrant and are significantly correlated with W1C, W3C, W5C, and W6S, primarily influenced by ammonia, alkanes, and aromatic compounds. Notably, despite HY and JDH being located in the second quadrant, their relative distance in the PCA space is considerable, indicating distinct differences in their VOC characteristics. Finally, FY is situated in the fourth quadrant and shows a correlation with W3S, highlighting its sensitivity to alkane compounds.

The data from the E-nose, collected through ten sensors including W1C, W5S, W3C, W6S, W5C, W1S, W1W, W2S, W2W, and W3S, illustrate the olfactory profiles of the four samples, as displayed in the radial diagram in Figure 1B. As shown in the figure, sensors W1W and W5S exhibited stronger responses to the samples compared to the other sensors, indicating that the olfactory profiles of strawberry scents were more reactive to sulfides, pyrazine compounds, and nitrogen oxides. In contrast, no sensor demonstrated sensitivity to esters, alcohols, ketones, and aldehydes present in the strawberry samples, which are crucial aroma compounds [6,7]. This further suggests that the E-nose lacks full sensitivity to certain aroma components found in strawberries, as it can only characterize a portion of aroma traits, thereby restricting its representational capabilities.

### 3.2. HS-GC-IMS Analysis in Four Strawberry Fruit Varieties

**Figure 2:**
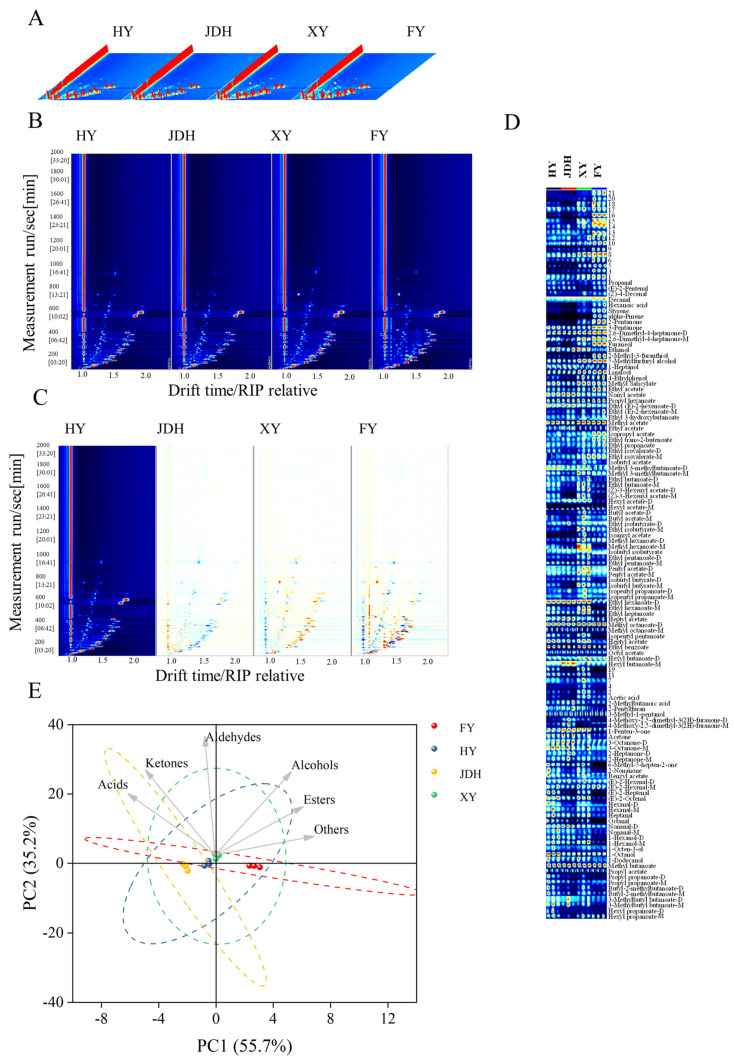
GC-IMS results for VOCs in the four strawberry types: (**A**) 3D spectrum; (**B**) direct correlation 2D diagram; (**C**) difference contrast 2D diagram; (**D**) fingerprint; (**E**) biplot. The numbers indicate the VOCs that were not detected using GC-IMS.

This study focused on VOCs from four distinct varieties of strawberries, utilizing the HS-GC-IMS database for analysis. As indicated in Table S1, a total of 109 compounds were identified, which included 2 alkenes, 3 acids, 11 alcohols, 15 ketones, 15 aldehydes, and 61 esters, along with other categories. To better illustrate the variations in signal peak intensities among the different strawberry types, a three-dimensional graphical evaluation was performed (Figure 2A). This diagram emphasized the various types and concentrations of VOCs found within each strawberry category. The X, Y, and Z axes represented the ion migration time, the retention time (RT) in the gas chromatograph, and the peak signal intensity for the quantitative analysis of VOCs, respectively. Although the categories of volatile aromas present across the four strawberry variants were comparable, the intensity of the signals exhibited differences. In Figure 2B, a two-dimensional representation provides a top-down view of the three-dimensional topographic map, illustrating the signal levels of diverse volatile aromas among the four strawberry types. By modifying both the ion drift timing and the positioning of the reactive ion peak (RIP), it was discovered that certain volatile compounds were located to the right of the RIP. The coloration of the ion peaks reflects the strength of the volatile components, showcasing a gradient from blue to red, where deeper shades indicate higher peak intensities. Most VOCs had retention times between 200 and 600 s, although a few were recorded with retention times extending from 800 to 1000 s.

To examine the VOCs present in four distinct strawberry varieties, the spectrum obtained for the HY strawberry (the most prevalent type cultivated in the Huaxi District of Guiyang City, as described in Section 2.1) was used as the baseline to subsequently deduce the reference spectrum of the other varieties. This approach allowed us to generate a comparative chart that highlighted the differences among the four strawberry samples. The resulting diagram, which depicts these variations, is presented in Figure 2C. When the concentration of a specific compound aligns with that of the reference strawberry sample, the background appears white. Conversely, a higher concentration of a VOC in the target sample is indicated by a red hue, while a lower concentration is represented by blue. The arrangement and intensity of these colors provide a visual representation of the disparities in VOCs. Notable changes in both the composition and concentration of volatile compounds among the four strawberry varieties are illustrated in Figure 2C.

To perform a quantitative analysis of the variations in VOCs, we utilized the GalleryPlot plug-in, which enabled us to generate a fingerprint illustrating the volatile compounds found in strawberries (Figure 2D). As shown in Figure 2D, the intensity of the color for each spot reflects the concentration of VOCs in different strawberry samples; a deeper red hue is associated with a greater concentration of the specific compound. Unknown substances are represented by numerical identifiers. Based on this criterion, several components, including hexyl butanoate, hexyl propanoate, 3-methylbutyl butanoate, isobutyl butyrate, hexyl acetate, isobutyl isobutyrate, isoamyl acetate, 1-dodecanol, 3-methyl-1-pentanol, nonanal, hexanal, benzaldehyde, styrene, 2-pentanone, 2-heptanone, 2-pentylfuran, and hexanoic acid exhibited greater relative abundance in HY strawberries compared to the other three types. Furthermore, JDH strawberries demonstrated a relatively higher concentration of VOCs, including (E)-2-heptenal, octanal, heptanal, 1-hexanol, acetone, 1-penten-3-one, and 2-methylbutanoic acid. Additionally, XY strawberries showed increased relative amounts of ethyl benzoate, methyl octanoate, ethyl heptanoate, butyl-2-methylbutanoate, (Z)-3-hexenyl acetate, methyl hexanoate, propyl propanoate, ethyl butanoate, methyl 3-methylbutanoate, propyl acetate, methyl butanoate, propyl hexanoate, methyl salicylate, 5-methylfurfuryl alcohol, 1-octanol, 1-octen-3-ol, furaneol, 3-octanone, 3-pentanone, 2-pentanone, 2,6-dimethyl-4-heptanone, alpha-pinene, (E)-2-octenal, (Z)-4-decenal, decanal, and propanal. Notably, FY strawberries contained relatively higher concentrations of VOCs than the other three varieties, which included octyl acetate, isopentyl pentanoate, benzyl acetate, heptyl acetate, ethyl hexanoate, isopentyl propanoate, pentyl acetate, butyl acetate, ethyl pentanoate, isoamyl acetate, ethyl isovalerate, isobutyl acetate, ethyl isobutyrate, ethyl propanoate, ethyl acetate, methyl acetate, ethyl trans-2-butenoate, ethyl 3-hydroxybutanoate, ethyl (E)-2-hexenoate, eonyl acetate, ethyl octanoate, 4-methoxy-2,5-dimethyl-3(2H)-furanone, 6-methyl-5-hepten-2-one, 4-ethylphenol, linalool, 1-heptanol, ethanol, 2-methyl-3-furanthiol, (E)-2-hexenal, and (E)-2-pentenal. This research indicated that, while the VOCs identified in the four varieties of strawberries were comparable, marked differences in their concentrations were observed.

Figure 2E systematically analyzes the correlation between various VOCs (such as ketones and acids) and four strawberry varieties by integrating the loading plot and score plot of PCA. The variance contribution rates of PC1 and PC2 were 55.7% and 35.2%, respectively, resulting in a cumulative explained variance of 90.9%. This indicates that the model effectively preserved the variability characteristics of the original data. The score plot revealed that FY could be distinctly differentiated from the other three strawberry varieties, while HY and XY exhibited similarities. Additionally, JDH could also be distinguished from the other varieties. The loading plot indicated that esters and other compounds were positioned on the positive half-axis of PC1 and were strongly correlated with the XY strawberry, suggesting that FY contained a higher concentration of ester compounds. Conversely, compounds such as 2-methylbutanoic acid and acetic acid were found on the negative half-axis of PC1 and were strongly correlated with JDH. However, FY and HY were situated close to the origin, indicating that their contributions to the two components were relatively small and might be more associated with other unrepresented components.

### 3.3. HS-SPME-GC-MS Evaluation in Four Strawberry Varieties

The volatile compounds present in four different kinds of strawberries were evaluated utilizing HS-SPME-GC-MS techniques. The analysis showed a total of 105 VOCs in these four varieties: 48 VOCs for HY, 56 VOCs for FY, 44 VOCs for JDH, and 60 VOCs for XY. Among these VOCs, there were 47 esters, 14 aldehydes, 8 ketones, 23 alkenes, 6 alcohols, 4 acids, and 3 alkanes (Table S2). Furthermore, 18 compounds were consistently identified across all four varieties, including methyl hexanoate, ethyl hexanoate, methyl octanoate, hexyl butanoate, (2E)-2-hexen-1-yl ester butanoic acid, methyl salicylate, isopentyl hexanoate, (Z)-hexanoic acid 3-hexenyl ester, hexyl hexanoate, (E)-β-farnesene, benzaldehyde, (E)-2-octenal, (E,E)-2,6-nonadienal, (E,E)-2,4-decadienal, linalool, (E)-nerolidol, and octanoic acid, as well as (E)-2-hexenyl hexanoate.

**Figure 3:**
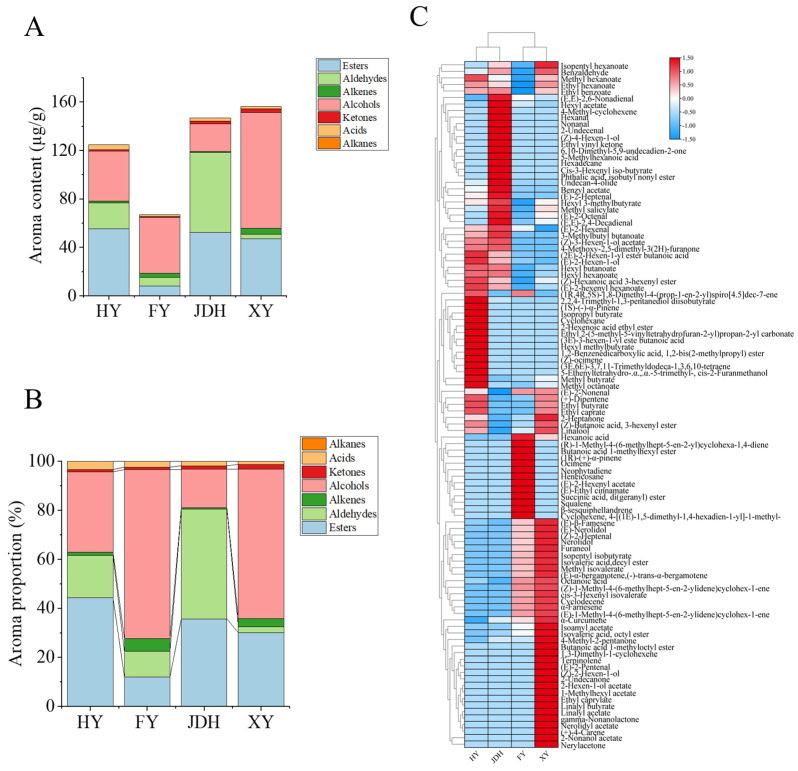
VOCs detected by GC-MS in the four strawberry types. (**A**) The relative contents of various types of VOCs; (**B**) the proportion of various types of VOCs; (**C**) heatmap of various types of VOCs.

As demonstrated in Figure 3A,B, the VOCs in XY strawberries showed the greatest diversity and abundance, where alcohols, esters, and alkenes made up 61.0%, 30.0%, and 3.2%, respectively. In terms of VOC types and amounts, JDH strawberries took the second spot, with their composition consisting of aldehydes, esters, and alcohols at 44.7%, 35.7%, and 15.7%, respectively. The HY variety presented esters, alcohols, and aldehydes in proportions of 44.2%, 32.9%, and 17.4%, respectively. On the other hand, the variety and quantity of VOCs in FY strawberries were the lowest, with alcohols, esters, and aldehydes making up 58.9%, 12.1%, and 10.3% of their composition, respectively.

Additionally, the analysis of clustering related to the heatmap (Figure 3C) suggested that the VOCs present in the deeper red area might function as crucial aroma components in differentiating and recognizing the four varieties of strawberries. As illustrated in Figure 3C, the XY strawberries exhibited the greatest diversity and abundance of VOCs. Specifically, (E)-nerolidol (60 µg/g), linalool (35 µg/g), and ethyl hexanoate (25 µg/g) were the predominant VOCs, constituting 76.6% of the total. On the other hand, JDH strawberries revealed higher levels of (E)-2-hexenal (44 µg/g), (E)-nerolidol (19 µg/g), hexyl acetate (19 µg/g), and ethyl hexanoate (16 µg/g), accounting for 73.3% of the total. For HY strawberries, the major VOCs included ethyl hexanoate (25 µg/g), linalool (21 µg/g), (E)-nerolidol (20 µg/g), and (E)-2-hexenal (12 µg/g), representing 62.4% of the total. Additionally, in FY strawberries, the main VOCs were (E)-nerolidol (35 µg/g), linalool (11 µg/g), and (E)-2-hexenal (5 µg/g), which accounted for 73.8% of the total.

As shown in Figure 3C and Table S2, HY strawberries exhibited 10 characteristic VOCs, including isopropyl butyrate, 2-hexenoic acid ethyl ester, ethyl 2-(5-methyl-5-vinyltetrahydrofuran-2-yl) propan-2-yl carbonate, hexyl methylbutyrate, 1,2-benzenedicarboxylic acid-1,2-bis(2-methylpropyl) ester, (1S)-(-)-α-pinene, (Z)-ocimene, (3E,6E)-3,7,11-trimethyldodeca-1,3,6,10-tetraene, 5-ethenyltetrahydro-α-α-5-trimethyl-cis-2-furanmethanol, and cyclohexane. Additionally, FY strawberries contained 12 characteristic VOCs, which included (E)-2-hexenyl acetate, butanoic acid 1-methylhexyl ester, (E)-ethyl cinnamate, succinic acid-di (geranyl) ester, (1R)-(+)-α-pinene, ocimene, squalene, (R)-1-methyl-4-(6-methylhept-5-en-2-yl) cyclohexa-1,4-diene, β-sesquiphellandrene, cyclohexene-4-[(1E)-1,5-dimethyl-1,4-hexadien-1-yl]-1-methyl, and neophytadiene, as well as heneicosane. Furthermore, JDH strawberries were characterized by 11 VOCs, including cis-3-hexenyl iso-butyrate, phthalic acid-isobutyl nonyl ester, 4-methyl-cyclohexene, hexanal, nonanal, 2-undecenal, (Z)-4-hexen-1-ol, ethyl vinyl ketone, 6,10-dimethyl-5,9-undecadien-2-one, 5-methylhexanoic acid, and hexadecane. Finally, XY strawberries exhibited 16 characteristic VOCs: 2-hexen-1-ol acetate, 1-methylhexyl acetate, ethyl caprylate, 2-nonanol acetate, butanoic acid 1-methyloctyl ester, linalyl butyrate, linalyl acetate, gamma-nonanolactone, nerolidyl acetate, (+)-4-carene, terpinolene, 1,3-dimethyl-1-cyclohexene, (E)-2-pentenal, (Z)-2-hexen-1-ol, 2-undecanone, and nerylacetone. The distinctive VOCs identified in these four different strawberry varieties were crucial for their discrimination and characterization and might serve as key biomarkers for these strawberry types.

In conclusion, XY strawberries exhibited the highest diversity and relative concentrations of VOCs, along with the most distinctive characteristic VOCs. This indicated that XY strawberries possessed a more complicated volatile aroma. Furthermore, each of the four strawberry varieties displayed unique characteristic VOCs, which could be used as key compounds for discriminating between strawberry varieties and could also be utilized as biomarkers for the detection of processed foods.

### 3.4. Key Aroma Component Analysis in Four Strawberry Fruit Varieties

The composition of VOCs does not always correspond directly with flavor; therefore, researchers often utilize the relative odor activity value (ROAV) to identify key aroma-active substances that affect the overall fragrance of a sample [28]. Human olfaction can detect VOCs with an ROAV (relative concentration/odor threshold) exceeding one, which are regarded as significant flavor components that shape the specific flavor profile of strawberries [25]. Among these four strawberry varieties, 24, 19, 21, and 27 VOCs with an ROAV exceeding 1 were detected in HY, FY, JDH, and XY, separately.

**Figure 4:**
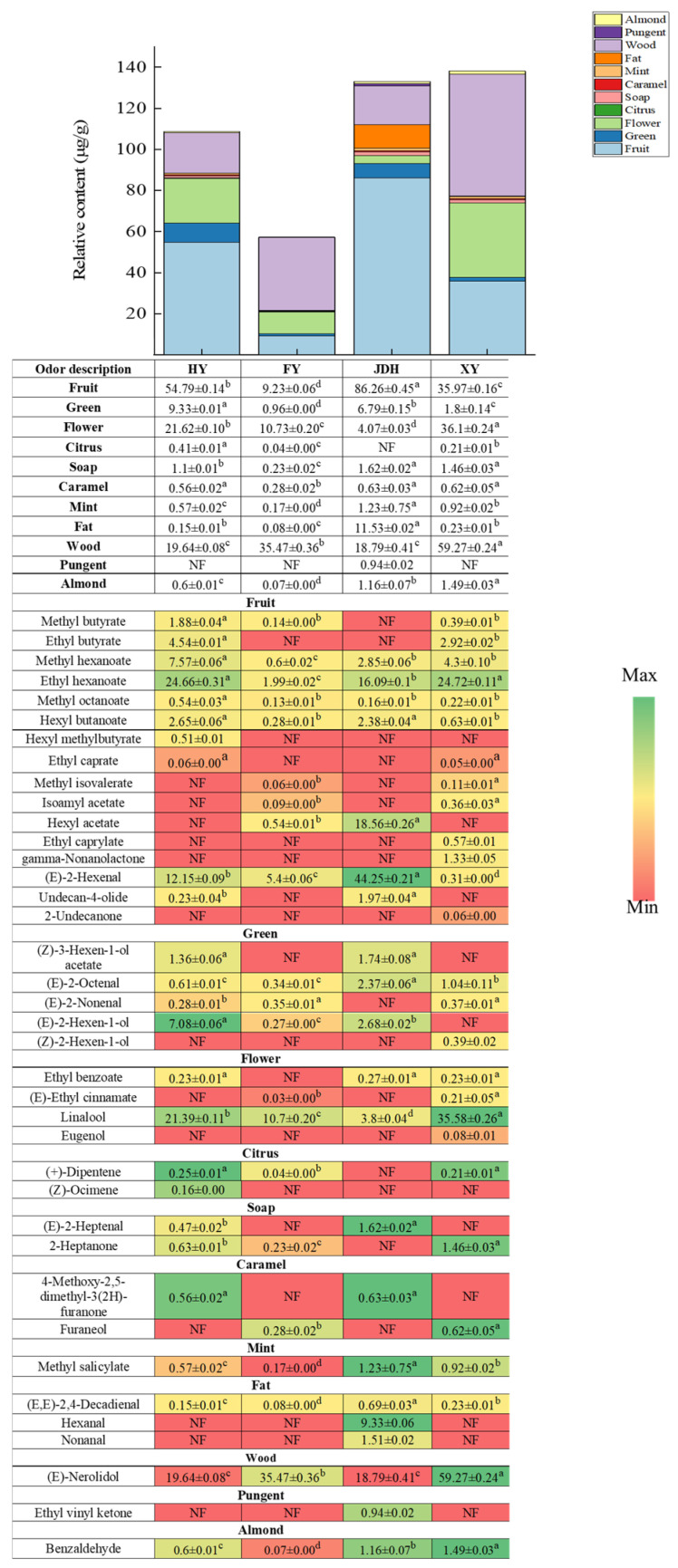
Identification and quantification analysis of the VOCs with an ROAV > 1 in the four kinds of strawberry fruits. ‘NF’ represents not found. a~d in the same row indicate that there is significant difference among these strawberry fruits varieties (*p* < 0.05).

As illustrated in Figure 4, we selected VOCs that had an ROAV exceeding 1 for a comparative examination of the fruits from four different strawberry varieties. The HY strawberry showed 11 unique aromas with an ROAV > 50, which were generated from ethyl butyrate, (E)-2-hexenal, methyl hexanoate, ethyl hexanoate, (Z)-3-hexen-1-ol acetate, (E)-2-octenal, linalool, (E)-2-nonenal, (E, E)-2,4-decadienal, (E)-nerolidol, and undecan-4-olide. The FY strawberry fruit demonstrated seven distinct aromas with an ROAV > 50, derived from ethyl hexanoate, (E)-2-octenal, linalool, (E)-2-nonenal, (E, E)-2,4-decadienal, (E)-nerolidol, and undecan-4-olide. The JDH strawberry fruit presented 11 different aromas with an ROAV > 50: hexanal, (E)-2-hexenal, ethyl hexanoate, (Z)-3-hexenyl acetate, hexyl acetate, (E)-2-octenal, linalool, nonanal, (E, E)-2,4-decadienal, (E)-nerolidol, and undecan-4-olide. The XY strawberry fruit also exhibited 11 unique aromas with an ROAV > 50: ethyl butyrate, isoamyl acetate, methyl hexanoate, ethyl hexanoate, (E)-2-octenal, linalool, (E)-2-nonenal, (E, E)-2,4-decadienal, eugenol, (E)-nerolidol, and gamma-nonanolactone. Among the four strawberry varieties, only four compounds, ethyl hexanoate, (E)-2-octenal, linalool, and (E)-nerolidol, were common, contributing to the sweet fruity, fresh green, floral citrusy, and woody aromas, respectively [29,30]. The significant differences in VOCs with an ROAV > 50 among the four strawberry varieties might account for the variation in their characteristic aromas.

The fruity aroma of strawberries is characterized by a blend of caramel, floral, fruity, fatty, and minty notes [31,32]. As shown in Figure 4, VOCs with an ROAV greater than 1 were selected for comparative analysis among four strawberry varieties. The HY strawberry variety exhibited ten distinct aromas: fruity, green, flowery, citrussy, soapy, caramel, minty, fatty, woody, and almond aromas. The FY strawberry variety displayed ten different aromas: fruity, green, flowery, citrussy, soapy, caramel, minty, fatty, woody, and almond aromas. The JDH strawberry variety had ten unique aromas: fruity, green, flowery, soapy, caramel, minty, fatty, woody, pungent, and almond aromas. Finally, the XY strawberry variety demonstrated ten distinct aromas, comprising fruity, green, flowery, citrussy, soapy, caramel, minty, fatty, woody, and almond aromas.

The XY strawberry contained 12 distinct varieties of VOCs attributed to its fruity odor, exceeding the 10 types found in HY strawberry, 9 types in FY strawberry, and 6 types in JDH strawberry. Additionally, the XY strawberry exhibited four unique VOCs associated with a flowery aroma, which was more than the two found in HY, one in FY, and three in JDH strawberries. Furthermore, the HY strawberry had four types of VOCs attributed to a green odor, which was greater than the three types present in FY, JDH, and XY strawberries. Moreover, the HY strawberry contained two types of VOCs related to a soapy odor, surpassing the individual type found in the other three varieties. Conversely, the JDH strawberry shared four VOCs associated with a fatty aroma, which was more than the single type identified in FY, JDH, and XY strawberries. Notably, all four strawberry flavors featured woody, minty, and caramel aromas. The specific citrussy odor in XY, FY, and HY strawberries was attributed to (+)-dipentene, which was absent from the JDH strawberry. Both XY and JDH strawberries exhibited an almond aroma, attributed to benzaldehyde. Importantly, the JDH strawberry displayed a pungent aroma provided by ethyl vinyl ketone, which was identified as a unique characteristic distinguishing this variety from the others.

The VOCs with a relative abundance of greater than one (ROAV > 1) in four different strawberry varieties were studied and compared. As shown in Figure 4, the XY strawberry exhibited the highest contents of VOCs related to flowery and woody odors in comparison with the other three types. The JDH strawberry ranked second in VOC levels, with notably higher fat and soap aromas than the other strawberries. The HY strawberry had the third-highest VOC levels, characterized by a pronounced green aroma. Conversely, the FY strawberry displayed the lowest VOC levels, with none of its flavors being particularly distinctive. Overall, these four strawberry varieties exhibited distinct VOC profiles that could serve as key biomarkers for differentiating between varieties, thereby assisting consumers in selecting the strawberries that best meet their preferences.

### 3.5. Comparison of Measurement Capabilities of Strawberry VOCs Between HS-GC-IMS and HS-SPME-GC-MS

**Figure 5:**
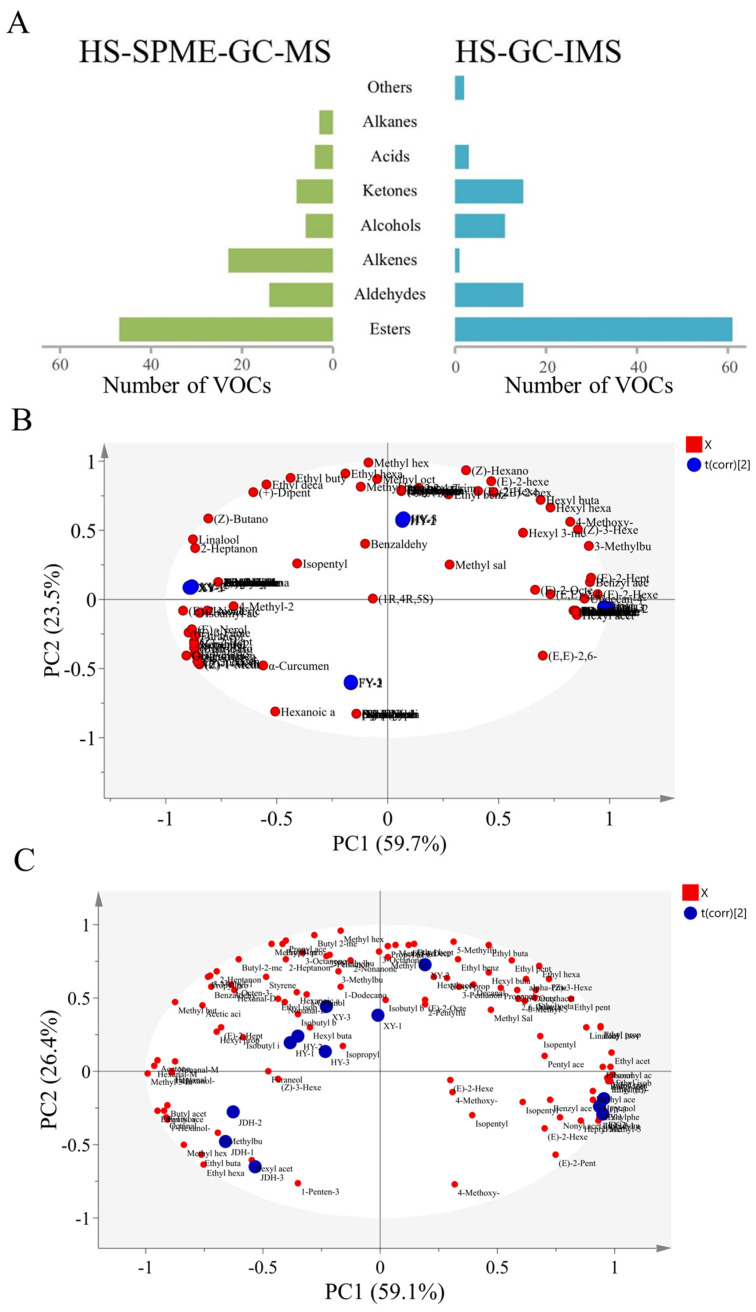
(**A**) Comparison of the various categories of VOCs detected through HS-GC-IMS and HS-SPME-GC-MS; (**B**) PCA diagram for HS-SPME-GC-MS results; (**C**) PCA diagram for HS-GC-IMS results.

The ability to measure VOCs varied between HS-GC-IMS and HS-SPME-GC-MS. HS-GC-IMS exhibited superior sensitivity for determining VOCs with reduced boiling points when in comparison with HS-SPME-GC-MS, which enhanced its proficiency in detecting highly volatile compounds [33]. As a result, HS-GC-IMS identified more low-boiling volatile components than HS-SPME-GC-MS. Nevertheless, both techniques uncovered similar VOC types in different strawberry samples, even though there were significant differences between the two. As illustrated in Figure 5A, 105 VOCs in total were measured through HS-SPME-GC-MS, while HS-GC-IMS revealed 109 VOCs. It was important to highlight that HS-GC-IMS identified a greater number of VOCs in comparison to HS-SPME-GC-MS, with the exception of alkenes, acids, and alkanes. This finding indicated that HS-SPME-GC-MS demonstrated superior efficiency in detecting alkenes, acids, and alkanes in strawberry fruits, whereas HS-GC-IMS proved to be more effective for the identification of esters, aldehydes, alcohols, ketones, and other categories of VOCs. Both techniques, HS-SPME-GC-MS and HS-GC-IMS, offered unique benefits in the detection of several VOCs.

To enhance the comparison of the two instruments’ capabilities in differentiating the VOCs present in strawberries, a PCA was employed on both datasets [34]. The resulting PCA score plots can be viewed in Figure 5B,C. Specifically, Figure 5B depicts the VOC content PCA scores for four distinct strawberry varieties, as analyzed using the HS-SPME-GC-MS technique. PC1 described 59.5% of the total variance, and PC2 contributed 23.3%, leading to an overall contribution rate of 82.8%. Noticeable variations were identified among the four strawberry varieties, which enabled a clear differentiation among them. Moreover, JDH exhibited interactions with various VOCs, including (E)-2-hexenal (odor: apple, green), benzyl acetate (odor: fresh, boiled vegetable), hexyl acetate (odor: fruity, herby), (E)-2-heptenal (odor: soapy, fatty, almond), undecan-4-olide (odor: soapy, fatty, almond), hexanal (odor: grassy, tallow, fatty), and ethyl vinyl ketone (odor: fishy, pungent). The aroma profile of the JDH strawberry variety was characterized by fruity, fresh, fatty, and pungent notes. In the case of HY strawberries, they exhibited VOCs such as methyl butyrate (odor: ether, fruity, sweet), methyl hexanoate (odor: fruity, fresh, sweet), ethyl hexanoate (odor: apple peel, fruity), methyl octanoate (odor: orange), hexyl methylbutyrate (odor: strawberry), and benzaldehyde (odor: almond, burnt sugar). The aroma of the HY strawberry variety showcased fruity and almond notes. For XY strawberries, VOCs included methyl isovalerate (odor: apple), isoamyl acetate (odor: banana), (E)-β-farnesene (odor: woody, citrussy, sweet), α-farnesene (odor: woody, sweet), (E)-2-nonenal (odor: cucumber, fatty, green), (E)-nerolidol (odor: woody, flowery, waxy), and furaneol (odor: caramel). The XY strawberry samples displayed aromatic characteristics with a combination of fruity, woody, and caramel notes. In contrast, FY strawberries did not exhibit any prominent aromatic features. Analyzing the aroma profiles of these varieties revealed that strawberries harbored a wide spectrum of fruity and fresh scents, alongside their distinctive aromatic traits.

The PCA score plots for the VOCs of four strawberry species analyzed via HS-GC-IMS are shown in Figure 5C. The overall variance was accounted for by PC1 and PC2, at rates of 59.1% and 26.4%, respectively. A noticeable separation among the samples was observed, demonstrating the ability of HS-GC-IMS to effectively differentiate the various strawberry varieties. The closeness between the HY and XY strawberries was particularly striking, consistent with the findings discussed in Section 3.2. Clearly, the four strawberry species exhibited significant variations in their aroma profiles across all experiments. Consequently, a beneficial approach would be to integrate these technologies for a comprehensive analysis of VOCs present in strawberries. In summary, the findings suggested that both the GC-MS and GC-IMS techniques effectively distinguished between different kinds of strawberry varieties. Although GC-IMS may not be able to match the quantification abilities of GC-MS for all peaks, it provides unique benefits, such as shorter durations for sampling and data analysis, along with increased sensitivity that allows for the detection of substances at low concentrations. Therefore, GC-IMS technology represents a quick and efficient option for aroma detection and shows significant promise in differentiating among various strawberry fruit varieties.

### 3.6. Discrimination of Four Strawberry Fruit Varieties Utilizing Both GC-MS and GC-IMS Technologies

**Figure 6:**
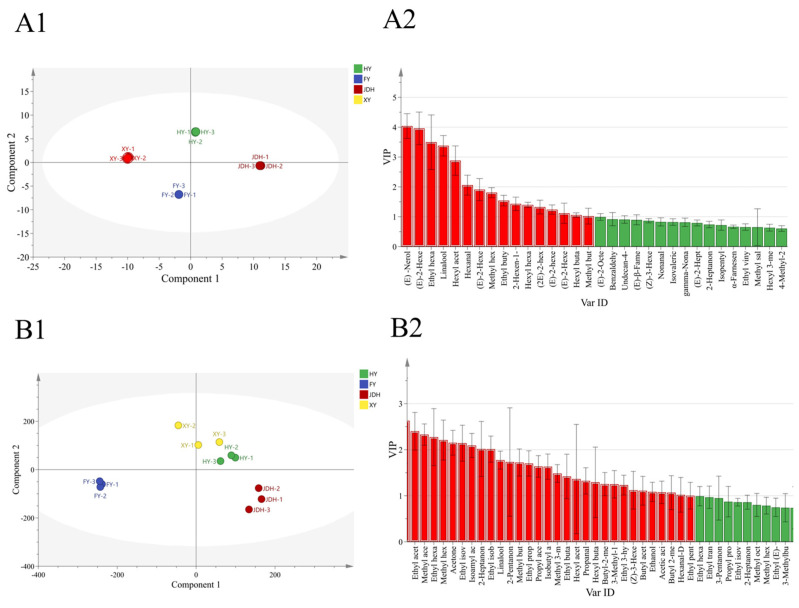
Orthogonal partial least squares–discriminant analysis (OPLS-DA) of HS-SPME-GC-MS data (R^2^X = 0.996, R^2^Y = 1, Q^2^ = 1) (**A1**) and HS-GC-IMS data (R^2^X = 0.934, R^2^Y = 0.964, Q^2^ = 0.921) (**B1**); VIP (variable importance in projection) results for HS-SPME-GC-MS data (**A2**) and HS-GC-IMS data (**B2**).

Orthogonal partial least squares discriminant analysis (OPLS-DA) employs a supervised analytical approach aimed at improving the visualization, discriminative analysis, and prediction of intricate datasets [35]. Unlike PCA, OPLS-DA focuses on enhancing the differences between groups based on pre-established categories, which leads to improved separation outcomes [36]. A correlation model may be created with OPLS-DA to associate the levels of VOCs, identified via HS-SPME-GC-MS, or the intensities of these VOCs, evaluated through HS-GC-IMS, with their corresponding sample categories [37]. To pinpoint the unique aroma compounds linked to the two OPLS-DA methods, the variable importance in projection (VIP) technique was employed, incorporating four strawberry varieties as variables in each model. Concerning variable X, the OPLS-DA model derived from HS-SPME-GC-MS included 105 VOCs in total, while the model based on HS-GC-IMS integrated 109 VOCs in total. The efficacy of discrimination for these models is depicted in Figure 6(A1,B1).

In the OPLS-DA scoring plot for HS-SPME-GC-MS (Figure 6A1), the total variance explained by the PC1 and PC2 is 59.7% and 23.5%, separately. Similarly, in the OPLS-DA scoring chart for HS-SPME-GC-IMS (Figure 6B1), the total variance is explained by the first and second components at rates of 59.1% and 26.4%, respectively. A larger separation between two samples on the score chart indicates a more significant difference between them, and, conversely, a smaller distance suggests lesser differentiation. The OPLS-DA graph presented in Figure 6(A1,B1) reveals a significant separation among the four varieties of strawberries. The scoring chart collected from HS-SPME-GC-MS clearly illustrates the distinct differences among these strawberry types. Similarly, the findings from the HS-GC-IMS scoring chart corroborate these results. To assess the performance of the OPLS-DA model, the R^2^X values for HS-SPME-GC-MS and HS-GC-IMS were found to be 0.996 and 0.934, respectively. The corresponding R^2^Y values were 1 and 0.964, while the Q^2^ values stood at 1 and 0.921, respectively. Following a series of 200 permutation tests, the validation of the OPLS-DA model confirmed that both R^2^ and Q^2^ values were above 0.5, suggesting a strong predictive capability and the absence of overfitting. Consequently, it was concluded that the model was effective in differentiating the four strawberry varieties.

To enhance our understanding of the differences in VOCs among the four strawberry varieties, the VOCs measured by HS-GC-IMS and HS-SPME-GC-MS underwent variable projection importance value (VIP) analysis. It is a widely acknowledged practice to utilize variables with a VIP value exceeding 1 for sample differentiation [38]. The VIP metric is regarded as a reflection of the significance of a variable in model development [38]. As depicted in Figure 6(A2,B2), the components analyzed by HS-SPME-GC-MS with VIP values over 1 included 10 esters, 3 alcohols, and 2 aldehydes, as follows: (E)-2-hexenal, (E)-neolidol, linalool, ethyl hexanoate, hexyl acetate, methyl hexanoate, (E)-2-hexen-1-ol, hexanal, ethyl butyrate, hexyl hexanoate, (E)-2-hexenyl hexanoate, (2E)-2-hexen-1-yl ester butanoic acid, hexyl butanoate, 2-hexen-1-ol acetate, and (E)-2-hexenyl acetate. The 15 VOCs were crucial in differentiating among the four varieties of strawberries. With regard to VOCs identified by HS-GC-IMS, the components with VIP values surpassing 1 mainly included 22 esters, 3 ketones, 3 alcohols, 2 aldehydes, and 1 acid. Notable examples were isopropyl acetate, ethyl acetate, methyl acetate, ethyl hexanoate-D (‘D’ represents dimer), methyl hexanoate-D, acetone, ethyl isovalerate-D, isoamyl acetate, 2-heptanone-D, ethyl isobutyrate-D, linalool, 2-pentanone, methyl butanoate, ethyl propanoate, propyl acetate, isobutyl acetate, methyl 3-methylbutanoate-D, ethyl butanoate-D, hexyl acetate-D, propanal, hexyl butanoate-M (‘M’ represents monomer), butyl-2-methylbutanoate-M, 3-methyl-1-pentanol, ethyl 3-hydroxybutanoate, (Z)-3-hexenyl acetate-D, butyl acetate-D, ethanol, acetic acid, butyl 2-methylbutanoate-D, hexanal-D, and ethyl pentanoate-D. These VOCs might serve as crucial indicators within the HS-GC-IMS OPLS-DA framework, aiding in the distinction between the four varieties of strawberries.

### 3.7. Correlation Analysis of Main Biomarkers and Aroma Characteristics of Strawberry

**Figure 7:**
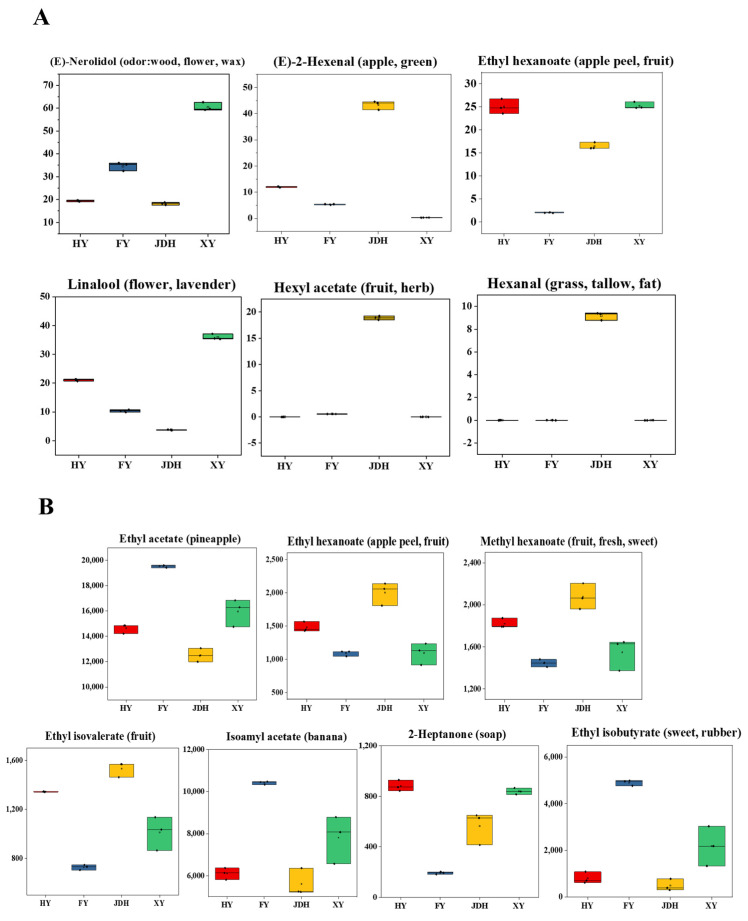
The box-type distribution of the principal aroma components in four strawberry fruit types ((**A**) HS-SPME-GC-MS; (**B**) HS-GC-IMS).

Figure 7 illustrates box plots representing odor compounds with variable importance in projection (VIP) values exceeding 2 across two OPLS-DA models. Specifically, Figure 7A showcases the box plots for (E)-neolidol, (E)-2-hexanal, ethyl hexanoate, linalool, hexanol, and hexanal. Significant differences were noted among the six groups regarding the six main biomarkers detected using GC-MS. The relative levels of (E)-nerolindol and linalool in XY strawberries were notably elevated compared to the other three strawberry types. These compounds might act as essential biomarkers for this particular group. The flavor descriptors associated with (E)-nerolindol and linalool elucidated the distinct flowery and woody aromas that defined the aroma profile of XY strawberries. For JDH strawberries, (E)-2-hexenal, hexyl acetate, and hexanal were pinpointed as vital biomarkers. The combined odor descriptors of these compounds suggested that JDH strawberries likely possessed more pronounced apple and grassy flavor notes. In HY strawberries, ethyl hexanoate was recognized as a key biomarker. Reflecting the olfactory properties associated with ethyl hexanoate, HY strawberries were characterized by a strong fruit aroma. In employing HS-SPME-GC-MS to differentiate among various strawberry varieties, the primary biomarkers that facilitated the distinction among the four categories included (E)-nerolindol, (E)-2-hexanal, ethyl hexanoate, linalool, hexyl acetate, and hexanal.

Figure 7B presents a box plot depicting seven aroma compounds identified by the OPLS-DA model of HS-GC-IMS, characterized by variable importance in projection (VIP) values greater than 2, along with their associated aroma descriptors. Notably, the concentration of 2-heptanone was notably elevated in HY strawberries, which might contribute a soap-like scent to their aroma profile. In FY strawberries, the concentrations of ethyl acetate, isoamyl acetate, and ethyl isobutyrate were comparatively high, producing aromas reminiscent of pineapple, banana, and various fruits; this suggested that the aroma profile of FY strawberries was heavily influenced by these tropical and fruity notes. Conversely, ethyl hexanoate, methyl hexanoate, and ethyl isovalerate could be regarded as significant biomarkers for JDH strawberries. Considering the aroma descriptors related to these biomarkers, it could be inferred that the aroma characteristics of JDH strawberries were likely to be reminiscent of apple peel, fresh fruit, and sweetness. While the relative concentrations of ethyl acetate, isoamyl acetate, and ethyl isobutyrate in HY strawberries were lower than those found in FY strawberries, this indicated that HY strawberries also possessed unique aromatic traits that evoked associations with pineapples, bananas, and various other fruits.

In summary, HS-SPME-GC-MS was capable of identifying six key biomarkers ((E)-nerolidol, (E)-2-hexenal, ethyl hexanoate, linalool, hexyl acetate, and hexanal), while HS-GC-IMS could detect seven essential biomarkers (ethyl acetate, ethyl hexanoate, methyl hexanoate, ethyl isovalerate, isoamyl acetate, 2-heptanone, and ethyl isobutyrate). It is important to highlight that the aroma compounds found in strawberries shared a common set of traits, such as fruity, fresh, sweet, and flowery notes. The next step involves investigating whether these compounds, identified during the screening process, are capable of distinguishing among the four different strawberry varieties through GC-O experiments.

## 4. Conclusions

In summary, the experiment demonstrated differences in VOCs across four types of strawberries by utilizing HS-GC-IMS, HS-SPME-GC-MS, and E-nose methodologies. The analysis with the E-nose indicated that the W5S and W1W sensors showed the highest response values for the four strawberry types, implying that these varieties were more responsive to sulfur compounds, pyrazines, and nitrogen oxides. Nevertheless, it was crucial to highlight that the E-nose was capable of distinguishing between the various strawberry types but could not fully identify the particular aroma compounds present within them. A total of 109 VOCs were identified using HS-GC-IMS, comprising 2 alkenes, 3 acids, 11 alcohols, 15 ketones, 15 aldehydes, 61 esters, and additional categories. Meanwhile, HS-SPME-GC-MS revealed 105 VOCs, which included 47 esters, 14 aldehydes, 8 ketones, 23 alkenes, 6 alcohols, 4 acids, and 3 alkanes. Among the varieties, the XY strawberry displayed the highest concentrations of VOCs associated with floral and woody scents when compared to the other three varieties. Following closely, the JDH strawberry came in second for VOC levels, notably having more pronounced fat and soap fragrances than the other strawberries. The HY strawberry ranked third, marked by a significant green scent. In contrast, the FY strawberry showed the lowest VOC levels, lacking any notably distinctive flavors. The methods HS-GC-MS and HS-SPME-GC-MS demonstrated effectiveness in distinguishing between four different varieties of strawberries, with OPLS-DA applied for additional analysis. In the case of HS-SPME-GC-MS, the OPLS-DA model identified six essential aroma components that differentiated these strawberry varieties: (E)-nerolidol, (E)-2-hexenal, ethyl hexanoate, linalool, hexyl acetate, and hexanal. Conversely, for HS-GC-IMS, the OPLS-DA model revealed seven important aroma compounds serving the same discriminative function: ethyl acetate, ethyl hexanoate, methyl hexanoate, ethyl isovalerate, isoamyl acetate, 2-heptanone, and ethyl isobutyrate. It was important to highlight that various aroma compounds in strawberries demonstrated comparable odor profiles. Employing these three methods for VOC detection in strawberries could deepen our insight into the aroma constituents present. Nonetheless, the mechanisms that govern the VOCs and their aromatic characteristics across the four varieties of strawberries have yet to be investigated. Ultimately, this research established a scientific foundation for recognizing the aroma compounds of four strawberry varieties cultivated in substrates and provided a holistic understanding of their aroma traits. A comprehensive identification of strawberry aroma compounds, utilizing HS-GC-IMS, HS-SPME-GC-MS, and E-nose technologies, may serve as a significant reference for growers and assist consumers in making informed purchasing choices.

## Data Availability

The original contributions presented in this study are included in this article, and further inquiries can be directed to the corresponding author.

## Supplementary Materials

The following supporting information can be downloaded at: https://www.mdpi.com/article/10.3390/foods14091464/s1, Figure S1: Pictures for four kinds of strawberries; Table S1: HS-GC-IMS results of VOCs in in the four strawberry varieties; Table S2: HS-SPME-GC-MS results of VOCs in the four strawberry varieties.
