# Supplementary material for: Discrimination and Characterization of the Aroma Profile in Four Strawberry Varieties Cultivated Under Substrates
Source: Foods. 2025 Apr 23;14(9):1464. doi: 10.3390/foods14091464 (PMC12071639; doi:10.3390/foods14091464)
Supplement: Supplementary file 1 [file foods-14-01464-s001.zip › foods-3571938-supplementary.pdf]

Supplementary Material

Pictures for four kinds of strawberries ("Hongyan HY", "Fenyu FY", "Xiangye XY", and "Jiandehong JDH"), volatile aroma components detected by HS-GC-IMS and HS-SPME-GC-MS in the four kinds of strawberry fruits.

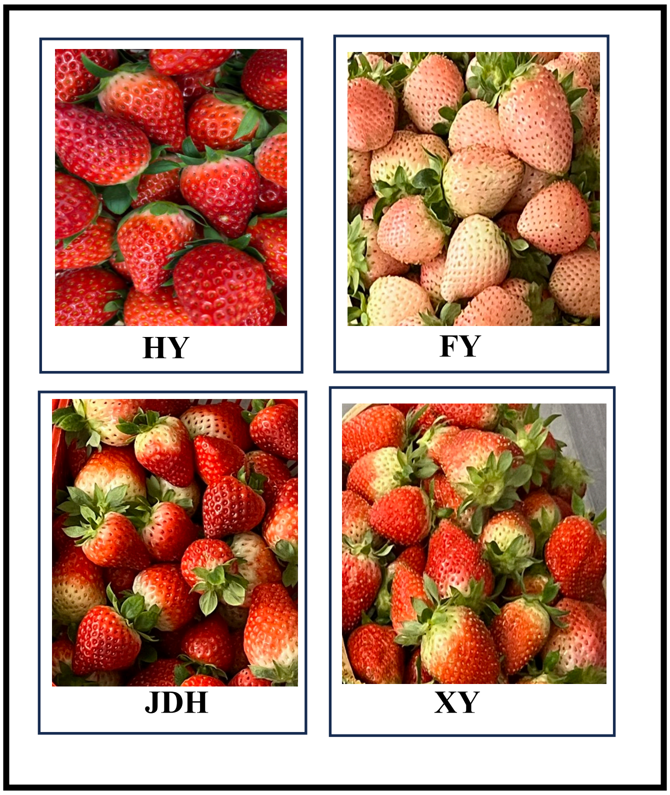

Fig. S1 Pictures for four kinds of strawberries

Table S1 HS-GC-IMS results of VOCs in in the four strawberry varieties

Volatile compound	CAS	RI	RT	HYGJ	FY	JDH	XY
Esters							
Octyl acetate	112-14-1	1277.5	1076.33	492.77±51.1	561.78±74.42	275.06±39.47	497.79±99.46
Hexyl butanoate-M	2639-63-6	1205.6	935.85	1640.74±331.56	1492.91±127.48	522.97±172.63	1429.92±323.56
Hexyl butanoate-D	2639-63-6	1204.8	934.24	216.36±80.46	89.59±10.24	92.21±4.16	97.64±11.67
Ethyl benzoate	93-89-0	1161.1	848.88	132.95±16.79	183.21±10.7	85.42±2.97	319.56±43.73
Isopentyl pentanoate	2050-9-1	1159.4	845.54	117.84±7.59	130.45±17.29	93.89±1.52	109.98±11.72
Benzyl acetate	140-11-4	1152.8	832.64	93.58±8.89	143.98±1.63	110.66±19.79	108.84±16.7
Methyl octanoate-M	111-11-5	1133	793.94	238.3±15.11	212.25±47.9	167.2±52.54	570.64±118.78
Heptyl acetate	112-06-1	1123.5	775.47	68.77±10.1	116.28±3.01	75.55±11.96	72.58±9.69
Hexyl propanoate-M	2445-76-3	1116.6	761.99	206.57±16.81	75.79±1.43	152.75±55.99	129.03±36.23
Hexyl propanoate-D	2445-76-3	1116.6	761.99	73.81±7.39	84.81±3.85	63.51±28.37	101.93±12.17
Ethyl heptanoate	106-30-9	1106.8	742.81	112.55±10.45	95.22±8.27	78.08±8.66	136.39±31.37
3-Methylbutyl butanoate-M	106-27-4	1074.4	677.56	609.02±100.43	313.34±39.19	285.78±92.99	510.06±111.33
3-Methylbutyl butanoate-D	106-27-4	1074	676.75	105.05±39.28	50.39±7.78	44.32±6.26	84.65±26.36
Butyl-2-methylbutanoate-M	15706-73-7	1060.3	648.55	1643.32±69.16	292.63±23.59	888.28±96.49	1640.22±42.32
Butyl 2-methylbutanoate-D	15706-73-7	1059.9	647.75	740.75±84.19	56.45±5.68	156.68±39.9	965.28±214.39
Hexyl acetate-M	142-92-7	1033.5	593.78	1771.83±13.34	1627.84±64.35	1989.34±265.23	1622.59±157.52
Hexyl acetate-D	142-92-7	1033.9	594.58	18836.32±564.12	18209.59±266.63	17397.11±446.7	18540.55±359.28
(Z)-3-Hexenyl acetate-M	1708-82-3	1024.9	576.05	348.08±34.89	340.39±10.28	377.54±28.19	366.06±40.88
(Z)-3-Hexenyl acetate-D	1708-82-3	1025.3	576.86	4044.32±215.9	4515.44±63.26	3420±217.46	4589.74±316.26
Ethyl hexanoate-M	123-66-0	1017.4	560.75	1479.2±61.11	1090.68±33.23	1999.19±141.64	1092.77±133.85
Ethyl hexanoate-D	123-66-0	1017.8	561.55	12105.67±152.99	13151±263.9	8752.86±816.47	13295.41±684
Isopentyl propanoate-M	105-68-0	983.3	496.45	207.97±7.83	295.8±33.16	273.42±53.27	229.12±69.26

Isopentyl propanoate-D	105-68-0	983.6	496.93	24.99±2.24	66.45±16.41	44.2±12.73	39.46±23.49
Isobutyl butyrate-M	539-90-2	968	471.396	481.88±74.4	331.86±9.73	250.14±46.2	318.45±67.53
Isobutyl butyrate-D	539-90-2	968.3	471.878	84.66±29.03	23.38±3.96	31.1±9.37	35.08±15.24
Methyl hexanoate-M	106-70-7	932.3	413.105	1820.15±38.38	1446.21±29.57	2076.75±100.48	1549.19±124.28
Methyl hexanoate-D	106-70-7	932.6	413.587	9495.25±163.12	7483.68±530.69	7446.94±1177	11023.7±631.33
Pentyl acetate-M	123-92-2	921.9	396.244	1058.99±25.62	898.04±12.19	1206.69±12.05	1040.04±76.07
Pentyl acetate-D	123-92-2	921.9	396.244	2983.86±424.21	3472.16±115.24	2693.62±199.74	3045.05±538.17
Ethyl pentanoate-M	539-82-2	910.1	376.974	782.7±36.75	802.6±52.9	603.79±47.96	836.71±45.93
Ethyl pentanoate-D	539-82-2	909.8	376.492	602.34±64.25	1198.55±176.84	285.05±54.02	1103.8±291.82
Isobutyl isobutyrate	97-85-8	906.9	371.675	214.76±35.87	128.88±5.01	167.49±17.29	169.6±22.3
Isoamyl acetate	123-92-2	889.3	344.697	6105.69±229.58	10420.06±64.82	5609.67±530.74	7808.63±926.46
Ethyl isovalerate-M	108-64-5	861	315.792	1343.91±2.23	726.68±17.24	1533.52±50.19	1011.07±112.31
Ethyl isovalerate-D	108-64-5	861.5	316.274	5259.79±225.38	8824.03±80.64	3778.23±474.72	7309.88±711.06
Propyl propanoate-M	106-36-5	826.4	280.386	837.83±30.91	401.41±23.18	683.63±106.3	813.34±39.73
Propyl propanoate-D	106-36-5	826.8	280.807	597.77±66.53	111.46±17.77	323.12±125.8	770.43±202.34
Butyl acetate-M	123-86-4	818.2	271.966	775.18±20.35	580.7±8.63	815.15±31.38	675.42±55.14
Butyl acetate-D	123-86-4	818.2	271.966	1169.34±55.03	2275.38±199.74	834.83±72.55	1281.25±262.83
Ethyl butanoate-M	105-54-4	804.6	258.072	654.15±9.89	547.2±4.98	815.43±69.94	592.01±64.55
Ethyl butanoate-D	105-54-4	805	258.493	8410.04±72.27	8411.35±94.29	7140.27±381.84	8973.37±287.25
Methyl 3-methylbutanoate-M	556-24-1	785.7	238.706	785.77±11.19	412.54±4.64	884.66±21.57	707.72±65.7
Methyl 3-methylbutanoate-D	556-24-1	786.5	239.548	2853.9±243.24	1454.34±33.34	2028.41±209.02	3400.59±297.55
Isobutyl acetate	110-19-0	779.1	233.654	538.81±88.64	3130.92±52.96	436.64±95.77	548.6±217.3
Ethyl isobutyrate-M	97-62-1	767.1	225.234	629.82±68.14	438.66±13.26	516.01±94.06	623.21±37.62
Ethyl isobutyrate-D	97-62-1	766.5	224.813	803.42±201.73	4911.92±99.47	492.38±207.03	2182.3±697.04
Propyl acetate	109-60-4	743.7	208.814	1873.54±277.05	353.22±91.92	879.45±113.02	2672.37±265.58
Methyl butanoate	623-42-7	728.7	198.289	7343.18±138.2	4432.88±125.29	7043.45±293.48	7357.45±282.12

Ethyl propanoate	105-37-3	716.7	189.869	3125.87±170.13	5008.49±23.12	1650.6±186.91	3755.41±551.76
Isopropyl acetate	108-21-4	674	164.608	7054.27±148.63	5082.06±224.6	4741.32±112.53	4575.61±231.6
Ethyl acetate	141-78-6	615.6	142.295	14643.21±304.16	19527.19±77.97	12515.5±436.1	15960.82±884.57
Methyl acetate	79-20-9	570.5	125.034	7630.6±86.26	10409.69±110.11	5708.03±42.54	6407.73±260.14
Ethyl trans-2-butenoate	10544-63-5	844.3	298.711	165.38±15.33	1147.22±110.65	114.4±23.71	487.45±113.92
Ethyl 3-hydroxybutanoate	5405-41-4	946.8	436.79	58.85±8.81	1606.63±115.01	40.45±3.69	118.14±32.78
Ethyl (E)-2-hexenoate-M	27829-72-7	1059.9	647.711	163.42±10.94	752.22±61.5	104.03±13.76	315.25±44.01
Ethyl (E)-2-hexenoate-D	27829-72-7	1059.9	647.711	61.55±3.2	275.53±42.09	30.88±1.91	105.04±23.81
Propyl hexanoate	626-77-7	1087.5	704.331	90.23±8.73	74.41±3.96	69.15±6.09	123.28±26.06
Methyl octanoate-D	111-11-5	1133.4	794.691	70.54±4.96	61.76±3.53	59.97±3.01	132.23±43.06
Nonyl acetate	143-13-5	1392.7	1301.531	222.51±14.61	257.76±6.87	229.63±8.79	228.57±4.23
Ethyl octanoate	106-32-1	1211.2	946.743	195.48±37.45	251.6±33.83	132.48±6.29	235.97±70
Methyl Salicylate	119-36-8	1227.5	978.601	60.38±6.01	82.07±8.3	64.75±20.3	87.61±14.1
Alcohols							
1-Dodecanol	112-53-8	1620.5	1746.55	899.8±175.67	660.33±32.37	603.49±68.13	758.18±105.25
Linalool	78-70-6	1111.4	751.82	852.57±122.49	672.32±68.77	201.97±22.58	274.68±14.48
1-Octanol	111-87-5	1090.2	709.78	62.92±5.13	3235.23±85.97	525.32±81.61	2649.39±194.65
1-Octen-3-ol	3391-86-4	998.9	522.89	65.51±1.21	53.81±3.98	82.99±5.49	133.21±23.58
1-Heptanol	111-70-6	993.9	513.79	131.7±16.98	41.45±1.74	59.69±1.68	76.55±9.94
5-Methylfurfuryl alcohol	3857-25-8	971.5	477.177	130.36±6.43	359.86±39.07	169.39±32.43	221.82±39.77
1-Hexanol-D	111-27-3	882.7	337.953	379.14±7.67	173.59±7.6	435.12±86.92	369.11±72.27
1-Hexanol-M	111-27-3	882.7	337.953	443.64±20.9	300.68±10.14	516.1±20.85	372.83±23.18
3-Methyl-1-pentanol	589-35-5	847.8	302.278	1994.97±142.11	545.64±12.73	1383.22±287.28	1923.5±224.25
Ethanol	64-17-5	516.4	104.368	1824.81±136.78	2938.38±66.63	1814.89±113.02	2383.51±69.3
2-Methyl-3-furanthiol	28588-74-1	895.4	352.967	88.59±13.78	286.69±17.47	98.06±8.91	99.9±22.64
Aldehydes							

Nonanal-M	124-19-6	1107.4	743.95	602.41±89.67	148.85±12.16	529.92±53.88	360.2±63.25
Nonanal-D	124-19-6	1107.4	743.95	109.69±19.01	64.89±1.44	75.04±15.89	84.62±9.59
(E)-2-Octenal	2548-87-0	1066.6	661.44	162.97±25.31	194.68±15.6	176.96±54.51	231.36±23.18
(E)-2-Heptenal	18829-55-5	955.6	451.163	207.84±31.98	126.94±4.73	229.68±75.73	222.89±31.32
Benzaldehyde	100-52-7	953.8	448.272	313.12±55.7	107.92±4.26	244.89±63.74	300.4±39.79
Heptanal	111-71-7	905.4	369.266	239.99±24.15	139.73±9.27	241.56±16	196.44±35.84
(E)-2-Hexenal-M	6728-26-3	847.8	302.303	599.14±51.61	968.52±30.22	746.02±91.33	715.75±62.55
Hexanal-M	66-25-1	797.2	250.494	554.82±16.85	315.7±7.97	588.15±58.23	477.68±34.04
Hexanal-D	66-25-1	797.6	250.915	794.33±95.75	50.1±0.86	295.3±114.13	456.72±133.96
(E)-2-Pentenal	1576-87-0	752.7	215.129	251.46±29.38	426.41±22.64	275.37±18.72	205.26±15.15
Propanal	123-38-6	519.4	105.489	893.88±45.77	1456.14±179.22	879.26±101.06	1880.16±210.7
(E)-2-Hexenal-D	6728-26-3	849.1	303.564	443.13±93.84	662.46±37.79	470.1±157.67	529.96±289.56
Octanal	124-13-0	1011.7	549.112	212.86±19.15	117.28±2.87	271.33±14.83	169.38±15.26
(Z)-4-Decenal	21662-09-9	1197.1	919.229	114.23±20.8	95.72±15.78	69.74±9.49	162.15±48.11
Decanal	112-31-2	1231.5	986.403	175.29±18.5	343.01±7.39	214.58±16.04	543.2±159
Ketones							
2-Nonanone	821-55-6	1097.7	725.08	85.49±11.83	50.37±1.95	40.97±5.15	68.51±7.4
6-Methyl-5-hepten-2-one	110-93-0	995.1	515.72	90.4±7.44	108.47±9.21	80.07±10.18	107.4±7.88
4-Methoxy-2,5-dimethyl-3(2H)-furanone-M	4077-47-8	1068.5	665.47	461.85±70.19	641.62±37.69	531.96±60.15	200.5±12.55
3-Octanone-M	106-68-3	998.1	521.27	358.61±20.89	266.08±4.56	302.11±13.17	458.88±49.11
3-Octanone-D	106-68-3	998.1	521.27	51.66±8.13	45.62±4.1	39.63±8.49	103.44±15.26
2,6-Dimethyl-4-heptanone-M	108-83-8	977.7	487.29	188.52±20.81	342.61±30.25	189.09±27.08	428.09±88.68
2-Heptanone-M	110-43-0	897.4	356.259	880.99±35.58	192.38±7.59	563.42±106.05	839.18±20.75
2-Heptanone-D	110-43-0	897.4	356.259	2852.74±435.9	277.64±7.64	515.17±126.28	2001.05±136.47
3-Pentanone	96-22-0	705.3	181.87	1937.42±164.06	2152.8±81.07	1946.98±122.28	2435.82±56.68
2-Pentanone	107-87-9	695	174.713	1292.47±146.3	441.28±22.83	773.45±82.16	2821.9±560.24

Acetone	67-64-1	535.9	111.801	5920.09±270.8	2754.39±69.86	7664.72±262.93	6383.91±137
2,6-Dimethyl-4-heptanone-D	108-83-8	978.6	488.727	33.52±0.82	138.7±29.09	30.48±4.4	175.66±100.91
4-Methoxy-2,5-dimethyl-3(2H)-furanone-D	4077-47-8	1068.6	665.526	93.37±23.29	94.73±9.86	73.12±8.68	63.81±4.04
1-Penten-3-one	1629-58-9	693.9	173.945	88.41±12	97.64±2.95	177.27±34.9	34.87±10.58
Furaneol	3658-77-3	1087.1	703.46	18.73±2.34	18.98±1.88	28.17±4.18	26.95±3.02
Acids							
2-Methylbutanoic acid	600-07-7	868.2	323.113	110.79±18.96	73±2.54	337.09±60.85	152.15±8.51
Acetic acid	64-19-7	589.8	132.399	915.88±44.09	400.86±32.72	1121.61±50.53	1309.57±38.69
Hexanoic acid	142-62-1	1033.3	593.332	400.77±3.35	325.43±14.07	347.71±58.95	365.04±34.71
Alkenes							
alpha-Pinene	2437-95-8	940.8	427.076	70.28±9.87	136.5±3.85	43.62±3.66	179.85±17.94
Styrene	100-42-5	901.3	362.522	231.36±52.71	94.18±8.05	128.8±14.45	202.18±23.04
Others							
2-Pentylfuran	3777-69-3	1010.7	547.05	221.75±22.33	197.15±29.48	160.16±8.82	207.08±16.41
4-Ethylphenol	123-07-9	1180.9	887.54	152.74±27.89	672.32±68.77	201.97±22.58	274.68±14.48

RI represents Retention Index; RT represents Retention Time. VOCs ending with '-M' and '-D' represent the Monomer and Dimer, respectively.

Table S2 HS-SPME-GC-MS results of VOCs in the four strawberry varieties

Volatile compound	RT	CAS	RI	Literature RI	HY (μg/g)	FY (μg/g)	JDH (μg/g)	XY (μg/g)
Esters								
Methyl butyrate	8.658	623-42-7	-	-	1.88656	0.140374	0	0.383143
Methyl isovalerate	10.267	556-24-1	-	766	0	0.059509	0	0.109229
Ethyl butyrate	11.039	105-54-4	808.69	-	4.546181	0	0	2.920679
Isopropyl butyrate	12.362	638-11-9	851.89	842	0.040464	0	0	0
Isoamyl acetate	13.409	123-92-2	886.09	876	0	0.093378	0	0.346071
Methyl hexanoate	14.742	106-70-7	935.50	936	7.552817	0.604311	2.780029	4.269749
Ethyl hexanoate	16.618	123-66-0	1011.04	1000	24.7542	1.967333	16.00511	24.85646
(Z)-3-Hexen-1-ol acetate	16.703	3681-71-8	1015.15	1007	1.353561	0	1.760563	0
Hexyl acetate	16.884	142-92-7	1023.91	1011	0	0.549553	18.50689	0
(E)-2-Hexenyl acetate	16.951	2497-18-9	1027.15	1017	0	2.03977	0	0
2-Hexen-1-ol acetate	16.951	10094-40-3	1027.15	-	0	0	0	4.091007
1-methylhexyl acetate	17.493	5921-82-4	1053.39	1043	0	0	0	0.091552
2-Hexenoic acid ethyl ester	17.56	1552-67-6	1056.63	1046	0.047499	0	0	0
3-Methylbutyl butanoate	17.808	106-27-4	1068.64	1054	0.157216	0	0.258478	0
Isopentyl isobutyrate	17.808	2050-1-3	1068.64	1021	0	0.249254	0	0.458323
Ethyl 2-(5-methyl-5-vinyltetrahydrofuran-2-yl)propan-2-yl carbonate	18.588	1000373-80-3	1107.22	-	0.089987	0	0	0
Methyl octanoate	19.121	111-11-5	1136.38	1126	0.505321	0.121462	0.158407	0.205778
Benzyl acetate	19.969	140-11-4	1182.77	1164	0.057929	0	0.211467	0
Ethyl benzoate	20.14	93-89-0	1192.12	1173	0.229906	0	0.25942	0.210308
(Z)-Butanoic acid, 3-hexenyl ester	20.226	16491-36-4	1196.83	1187	0.036481	0.012565	0	0.041042

(3E)-3-hexen-1-yl este butanoic acid	20.273	53398-84-8	1199.40	-	0.051484	0	0	0
Cis-3-Hexenyl iso-butyrate	20.273	41519-23-7	1199.40	1145	0	0	0.078844	0
Hexyl butanoate	20.359	2639-63-6	1203.92	1191	2.588297	0.287656	2.327945	0.634085
(2E)-2-Hexen-1-yl ester butanoic acid	20.407	53398-83-7	1206.43	1193	4.083392	0.740321	2.27437	0.696072
Ethyl caprylate	20.445	106-32-1	1208.41	1204	0	0	0	0.567461
Methyl salicylate	20.683	119-36-8	1220.85	1194	0.563219	0.172935	1.783831	0.901697
Butanoic acid 1-methylhexyl ester	20.74	39026-94-3	1223.82	-	0	0.0132	0	0
2-Nonanol acetate	21.159	14936-66-4	1245.72	-	0	0	0	0.069663
cis-3-Hexenyl isovalerate	21.216	35154-45-1	1248.69	1240	0	0.036063	0	0.056305
Hexyl methylbutyrate	21.225	10032-15-2	1249.16	1236	0.519666	0	0	0
Hexyl 3-methylbutyrate	21.302	10032-13-0	1253.19	1245	0.481599	0	0.918294	0.45641
Isopentyl hexanoate	21.463	2198-61-0	1261.60	1260	0.491774	0.307737	0.751336	1.20589
(Z)-Hexanoic acid 3-hexenyl ester	24.243	31501-11-8	1393.42	1380	0.045388	0.003604	0.032055	0.023139
Hexyl hexanoate	24.329	6378-65-0	1397.35	1386	4.284172	0.256962	4.060032	0.90529
Ethyl caprate	24.52	110-38-3	1405.14	1396	0.064948	0	0	0.054147
Butanoic acid 1-methyloctyl ester	24.767	69727-42-0	1414.67	-	0	0	0	0.173719
Linalyl butyrate	25.234	78-36-4	1432.70	1422	0	0	0	0.088553
Isovaleric acid, octyl ester	25.605	7786-58-5	1447.03	1440	0	0.360007	0.090162	1.557048
(E)-Ethyl cinnamate	26.662	4610-69-9	1487.84	1373	0	0.025127	0	0
2,2,4-Trimethyl-1,3-pentanediol diisobutyrate	29.87	6846-50-0	1618.65	-	0.543903	0	0.052533	0
Linalyl acetate	29.918	115-95-7	1621.00	1258	0	0	0	0.072288
Succinic acid, di(geranyl) ester	29.975	1000391-21-7	1623.80	-	0	0.022883	0	0
Isovaleric acid,decyl ester	30.508	1000340-25-8	1649.95	-	0	0.042127	0	0.07117
gamma-Nonanolactone	31.584	104-61-0	1703.23	1361	0	0	0	1.30801

Nerolidyl acetate	32.06	2306-78-7	1732.24	-	0	0	0	0.076228
1,2-Benzenedicarboxylic acid, 1,2-bis(2-methylpropyl) ester	34.916	84-69-5	1893.51	1873	0.179351	0	0	0
Phthalic acid, isobutyl nonyl ester	34.916	1000309-04-4	1893.51	-	0	0	0.105456	0
Alkenes								
(+)-4-Carene	17.113	29050-33-7	1035.00	1022	0	0	0	0.056907
(+)-Dipentene	17.379	5989-27-5	1047.87	-	0.245456	0.038253	0	0.209127
(1R)-(+)-α-pinene	17.446	7785-70-8	1051.11	-	0	0.029849	0	0
(1S)-(-)-α-Pinene	17.455	7785-26-4	1051.55	-	0.102489	0	0	0
(Z)-ocimene	17.684	3338-55-4	1062.63	1043	0.164446	0	0	0
Terpinolene	18.598	586-62-9	1107.77	1087	0	0	0	0.048055
4-Methyl-cyclohexene	18.921	591-47-9	1125.44	-	0	0	0.16457	0
Ocimene	19.264	7216-56-0	1144.20	-	0	0.22002	0	0
1,3-Dimethyl-1-cyclohexene	22.52	2808-76-6	1314.71	-	0	0	0	0.014837
(E)-β-Famesene	26.214	18794-84-8	1470.54	1459	0.754497	1.633235	0.603676	2.856363
Squalene	26.405	111-02-4	1477.92	2833	0	0.058545	0	0
(1R,4R,5S)-1,8-Dimethyl-4-(prop-1-en-2-yl)spiro[4.5]dec-7-ene	26.757	729602-94-2	1491.51	-	0.016978	0.015001	0	0
α-Curcumene	27.147	644-30-4	1506.76	1484	0	0.024153	0.017365	0.042597
(E)-α-bergamotene,(-)-trans-α-bergamotene	27.214	13474-59-4	1509.43	1439	0	0.26145	0	0.496472
(3E,6E)-3,7,11-Trimethyldodeca-1,3,6,10-tetraene	27.614	502-61-4	1525.35	1509	0.246452	0	0	0
α-Farnesene	27.614	502-61-4	1525.35	1509	0	0.66285	0	1.017251
(R)-1-Methyl-4-(6-methylhept-5-en-2-yl)cyclohexa-1,4-diene	27.899	28976-67-2	1536.69	1512	0	0.028958	0	0
(Z)-1-Methyl-4-(6-methylhept-5-en-2-ylidene)cyclohex-1-ene	28.099	13062-00-5	1544.65	-	0	0.081891	0	0.105447
β-Sesquiphellandrene	28.252	20307-83-9	1550.74	1522	0	0.158787	0	0
(E)-1-Methyl-4-(6-methylhept-5-en-2-ylidene)cyclohex-1-ene	28.471	53585-13-0	1559.45	1530	0	0.08066	0	0.113602

Cyclohexene, 4-[(1E)-1,5-dimethyl-1,4-hexadien-1-yl]-1-methyl-	28.633	25532-79-0	1565.90	1508	0	0.158443	0	0
Cyclodecene	30.099	3618-12-0	1629.88	-	0	0.038691	0	0.055556
Neophytadiene	34.202	504-96-1	1853.19	1837	0	0.018448	0	0
Aldehydes								
(E)-2-Pentenal	9.62	1576-87-0	-	765	0	0	0	0.305836
Hexanal	11.01	66-25-1	807.74	802	0	0	9.342881	0
(E)-2-Hexenal	12.781	6728-26-3	865.58	854	12.15648	5.43956	44.07646	0
(E)-2-Hexen-1-ol	13.162	928-95-0	878.02	862	7.09801	0.280014	2.692771	0
(E)-2-Heptenal	15.608	18829-55-5	969.39	958	0.474288	0	1.622518	0
(Z)-2-Heptenal	15.608	57266-86-1	969.39	964	0	0.193147	0	0.388141
Benzaldehyde	15.808	100-52-7	977.22	979	0.616536	0.06842	1.1687	1.497979
(E)-2-Octenal	17.903	2548-87-0	1073.23	1064	0.59639	0.327345	2.322061	0.995782
Nonanal	18.788	124-19-6	1118.16	1095	0	0	1.506601	0
(E,E)-2,6-Nonadienal	19.74	17587-33-6	1170.24	-	0.095146	0.146266	0.214554	0.126347
(E)-2-Nonenal	19.845	18829-56-6	1175.98	1165	0.276745	0.359156	0	0.368022
(E,E)-2,4-Decadienal	22.454	25152-84-5	1311.69	1314	0.138416	0.077305	0.699992	0.239953
2-Undecenal	23.929	2463-77-6	1382.32	1359	0	0	0.219645	0
Undecan-4-olide	31.574	104-67-6	1702.66	1589	0.21069	0	1.915783	0
Alcohols								
(Z)-2-Hexen-1-ol	13.219	928-94-9	879.88	865	0	0	0	0.394392
(Z)-4-Hexen-1-ol	16.703	928-91-6	1015.15	879	0	0	0.564934	0
5-ethenyltetrahydro-.α.,.α.-5-trimethyl-, cis-2-Furanmethanol	18.284	5989-33-3	1091.67	1075	0.070093	0	0	0
Linalool	18.779	78-70-6	1117.67	1101	21.32934	10.7691	3.828172	35.32593
Nerolidol	28.899	7212-44-4	1576.48	1562	0	0.064802	0	0.129774
(E)-Nerolidol	29.08	40716-66-3	1583.68	1567	19.64228	35.33596	18.72422	59.5759

Ketones								
Ethyl vinyl ketone	7.706	1629-58-9	-	678	0	0	0.948629	0
4-Methyl-2-pentanone	9.144	108-10-1	-	741	0	0.138764	0.157882	0.787059
2-Heptanone	13.818	110-43-0	899.44	893	0.638236	0.220924	0	1.467545
4-Methoxy-2,5-dimethyl-3(2H)-furanone	17.96	4077-47-8	1075.99	1051	0.555611	0	0.625617	0
Furaneol	18.112	3658-77-3	1083.35	1060	0	0.284897	0	0.610798
2-Undecanone	22.377	112-12-9	1308.18	1291	0	0	0	0.056926
6,10-Dimethyl-5,9-undecadien-2-one	26.109	689-67-8	1466.49	1456	0	0	0.398915	0
Nerylacetone	26.119	3879-26-3	1466.87	1445	0	0	0	0.163284
Acids								
Hexanoic acid	16.703	142-62-1	1015.15	1001	0	0.565981	0	0.249343
5-Methylhexanoic acid	17.436	628-46-6	1050.63	-	0	0	0.126423	0
Octanoic acid	19.969	124-07-2	1182.77	1178	0.265255	0.598436	0.160649	0.737705
(E)-2-hexenyl hexanoate	24.386	53398-86-0	1399.95	1391	3.795613	0.464832	2.39113	1.021766
Alkanes								
Cyclohexane	7.221	110-82-7	-	-	0.139978	0	0	0
Hexadecane	24.634	544-76-3	-	-	0	0	0.069255	0
Heneicosane	31.984	629-94-7	-	-	0	0.012109	0	0

RT represents Retention Time; RI represents Retention Index. Literature RI represents Retention Index calculated by C_7 ~ C_{40} n-alkanes on the HP-5 MS Capillary GC Column (Length 60 m, inner diameter 0.25 mm, and film thickness 0.25 µm; Agilent, USA); Literature RI was collected from NIST Chemistry WebBook database: webbook.nist.gov.
